# Decoherence and control of a qubit in spin baths: an exact master equation study

**DOI:** 10.1038/s41598-018-19977-9

**Published:** 2018-01-24

**Authors:** Jun Jing, Lian-Ao Wu

**Affiliations:** 1 Department of Physics, Zhejiang University, Hangzhou, 310027 Zhejiang, China; 2 Department of Theoretical Physics and History of Science, The Basque Country University (EHU/UPV), PO Box 644, 48080 Bilbao, Spain; 3 Ikerbasque, Basque Foundation for Science, 48011 Bilbao, Spain

## Abstract

In spin-based nanosystems for quantum information processing, electron spin qubits are subject to decoherence due to their interactions with nuclear spin environments. In this paper, we present an exact master equation for a central spin-1/2 system in time-dependent external fields and coupled to a spin-half bath in terms of hyperfine interaction. The master equation provides a unified description for free and controlled dynamics of the central spin and is formally independent of the details and size of spin environments. Different from the previous approaches, the master equation remains exact even in the presence of external control fields. Using the parameters for realistic nanosystems with nonzero nuclear spins, such as GaAs, we investigate the Overhauser’s effect on the decoherence dynamics of the central spin under different distributions of bath-spin frequencies and system-bath coupling strengths. Furthermore, we apply the leakage elimination operator, in a nonperturbative manner, to this system to suppress the decoherence induced by hyperfine interaction.

## Introduction

Understanding the nonequilibrium dynamics of quantum systems interacting with a large number of uncontrollable degrees of freedom is a rapidly emerging topic, developed in various fields, such as quantum optics and quantum devices based on mesoscopic and nanoscale solid systems^1,2^. The essential recipe for making such quantum devices is quantumness such as quantum correlation^3^ and coherence, which is often ruined by environmental noise^4^. Hence, defeating decoherence and restoring quantumness pose critical challenges to quantum technologies^5^, especially to quantum control practices.

In spin-based systems, quantum-computing proposals using quantum dots (QDs)^6,7^ have led to intensive studies on coherent control of quantum degrees of freedom, in particular electron spins. Technically, electron spins can be manipulated via external controls and have been used as qubits, while nuclear spins behave as a magnetic environment with spatial-fluctuation that causes decoherence of these qubits. Electron spin qubits in nuclear spin environments have three distinct merits: scalability, compatibility with semiconductor technology, and controllability due to its natural non-Markovianity. The last one will be responsible for a long decoherence time of the system and thus will be helpful to the focus of this study: nonperturbative control of the electron spin. For one of these spin qubits, the electron wave function is localized inside the nanoscale region and the nuclear spins coupled to the central spin form a nanoscale spin bath, which is dramatically different from the conventional Markovian bosonic bath. Therefore a full quantum description for the spin bath model is crucial for the fundamental study of both open quantum systems^8^ and the techniques or strategies of quantum control problems^9,10^. A variety of quantum theories^11^ for the free dynamics of a central spin in a spin bath have been developed, including the pair-correlation approximation, cluster expansion, linked-cluster expansion and cluster-correlation expansion.

For solid spin-qubit systems, a variety of mechanisms have been identified to be responsible for the electron spin decoherence, such as the spin-orbital scattering with phonons^12^, spectral diffusion due to dipolar interaction of nuclear spins^13^, and the hyperfine interaction between electron spin and environmental nuclear spins^14^. Low temperature (for confined electron spin in gate-defined QDs can be controlled electrically at <1K) and moderate magnetic (>0.1T) field^15^ can be used to exclude most of the noise mechanisms of the electron spin, including the pure dephasing due to the diagonal hyperfine interaction, but fails to suppress the decoherence due to off-diagonal part of the hyperfine interaction. This regime is the main focus of the present work. Under the hyperfine interaction, the most relevant noise sources for the central spins in quantum technologies, the nuclear spin bath is highly non-Markovian and the central electron spin in a quantum dot has a comparatively long coherence time^16–19^. To formulate these interesting observations, an exact master equation for the central electron spin in spin environment, in analogy to that for the spin in bosonic bath^20^, is desired for future QD quantum computer based on full polarization of nuclear spins. This equation also sets up a benchmark for evaluating the validity of existing perturbative approaches to the central spin in a spin environment, including the conventional second-order master equation, and is a perfect tool in checking the performance of nonperturbative quantum control strategies. Although we employ the same time-convolutionless (TCL) method as some previous works^21–23^ which yield second-order master equations, our derivation gives rise to an exact master equation. More importantly, our master equation allows us to perform the nonperturbative leakage elimination operator (LEO) protocol such that the formation of equation of motion under control remains the same as that for free evolution, while other approaches^24–26^ in the decoherence of electron spins are *no longer* exact in the presence of a time-dependent LEO operator on the free evolution.

This paper first derives an exact master equation for the central spin under the hyperfine interaction with environmental spins. This equation enables us to study the dynamics of the central spin, and more interestingly it demonstrates the control process using the leakage elimination operator protocol^27^. It is worth pointing out that this protocol is different from the existing dynamical control methods in spin baths, such as Hahn echo control and Carr-Purcell-Meiboom-Gill (CPMG) control^28^, which are based on the assumption that the central spin state is frozen within the control-pulse-applied durations and therefore are essentially of perturbative methods. The nonperturbative LEO protocol was introduced recently^29^ to suppress decoherence due to the presence of environmental interference. The advantage of the protocol is that we can introduce an LEO into the system Hamiltonian while keeping the open system dynamics exactly solvable irrespective to the size of the system. Because of the exact solvability, this master equation can help us to correctly understand the controllability of non-Markovianity and clarify possible confusion owing to the Markovian approximations such as the conventional Wigner-Weisskopf approximation. Furthermore, by using our exact master equation it is clearly shown that an ideal Markovian process is *not* controllable, which has been missing in the conventional Wigner-Weisskopf approximation.

## Exact master Equation

In semiconductor materials, the central electron spin and its spin bath are quantum objects and are subject to quantum descriptions. The nuclear spins in some systems like ^29^Si are of spin-1/2. In this section, we consider a central spin-1/2 embedded in a spin-half bath through the hyperfine interaction:

1 $${H}_{{\rm{tot}}}={\omega }_{0}{S}_{z}+\sum _{k}{\omega }_{k}{I}_{k}^{z}+\sum _{k}\frac{{A}_{k}}{2}({S}_{+}{I}_{k}^{-}+{S}_{-}{I}_{k}^{+})+\sum _{k}{A}_{k}{S}_{z}{I}_{k}^{z},$$

where *ω*_0_ and *ω*_*k*_ correspond to the Zeeman energy of the central and *k*-th environmental spins, *A*_*k*_ is the coupling strengths between the central spin and the *k*-th environmental spin, the operators *S* and *I* indicate the central spin and environmental spins, respectively. Concretely, *S*_*z*_ ≡ (|1〉〈1| − |0〉〈0|)/2, *S*_+_ ≡ |1〉〈0|, and *S*_−_ ≡ |0〉〈1|, where |1〉 (|0〉) is the upper (down) state of the central spin. This model applies to decoherence induced by nonzero nuclear spins, when the remaining sources of central spin relaxation and dephasing, e.g., spin-orbit scattering with phonons, are strongly suppressed by low temperatures and high magnetic fields. This can also be a typical spin-bath model characterizing physical entities such as atomic, molecular systems and artificial two-level systems.

Particularly, in GaAs quantum dot systems, *ω*_0_ is determined by the electron spin Zeeman effect of external magnetic field. The third and the fourth terms in Eq. (1) are termed as the flip-flop interaction and (longitudinal) Overhauser’s field, giving rise to inhomogeneous broadening and dephasing, respectively. The strength *A*_*k*_ is determined by the electron density at the site of nuclei. Typically, when the external magnetic field is in the order of 1T, the magnitude of the parameters satisfy *ω*_0_ ≈ ∑_*k*_*A*_*k*_ ≈ 10^3^ *ω*_*k*_. And the dipolar interaction between two nuclei separated by 1 nm distance is almost 10^−8^ *ω*_0_^30^ (so it is reasonable to ignore this interaction in the total Hamiltonian, especially in the short time limit). In the interaction picture with respect to the Hamiltonian ${H}_{0}={\omega }_{0}{S}_{z}+{\sum }_{k}({\omega }_{k}-{A}_{k}\mathrm{/2)}{I}_{k}^{z}$, the total Hamiltonian becomes

2 $${H}_{{\rm{tot}}}^{I}={e}^{i{H}_{0}t}({H}_{{\rm{tot}}}-{H}_{0}){e}^{-i{H}_{0}t}={S}_{+}(t){B}^{-}(t)+{S}_{-}(t){B}^{+}(t)+\mathrm{|1}\rangle \langle \mathrm{1|}{B}^{z},$$

where

3 $${B}^{\pm }(t)\equiv \sum _{k}\frac{{A}_{k}}{2}{I}_{k}^{\pm }{e}^{\pm i({\omega }_{k}-{A}_{k}\mathrm{/2)}t},\quad {S}_{\pm }(t)={S}_{\pm }{e}^{\pm i{\omega }_{0}t},\quad {B}^{z}\equiv \sum _{k}{A}_{k}{I}_{k}^{z}\mathrm{.}$$

Due to the commutation relation [*H*_t*ot*_, *S*_*z*_ + ∑_*k*_*I*_*z*_] = 0, the total exciton number or angular momentum is conserved under the total Hamiltonian, such that one can work in one of invariant subspaces with a given exciton number. It is interesting to note that, the transversal hyperfine term *S*_+_ (*t*)*B*^−^ (*t*) + *h*.*c*. results in the off-resonant transitions between the system and environmental spins, while the longitudinal hyperfine term $|1\rangle \langle 1|{B}^{z}$ provides additional contributions to the energy splitting. After a straightforward derivation (see Method), we can obtain an exact TCL master equation in the interaction picture

4 $${\partial }_{t}\rho (t)=\,-\frac{i}{2}\varepsilon (t)[{S}_{+}{S}_{-},\rho (t)]+\gamma (t)[{S}_{-}\rho (t){S}_{+}-\frac{1}{2}\{{S}_{+}{S}_{-},\rho (t)\}],$$

where $\varepsilon (t)\equiv \,-2{\rm{Im}}[\dot{G}(t)/G(t)]$ and $\gamma (t)\equiv \,-2{\rm{Re}}[\dot{G}(t)/G(t)]$. The first term in Eq. (4) attributes to the stark-shift effect and the second term is the source of decoherence. Both of the time-dependent coefficients *ε*(*t*) and *γ*(*t*) are determined by the imaginary and real parts of *Ġ*(*t*)/*G*(*t*), respectively. Serving as a propagator, the function *G*(*t*) is defined in Eq. (33), which is closely related to the two-point correlation function of the spin reservoir *f*(*t* − *s*) as defined in Method:

5 $$f(t-s)=\sum _{k}{(\frac{{A}_{k}}{2})}^{2}{e}^{i({\omega }_{0}-{\omega }_{k}+\frac{{A}_{k}}{2})(t-s)}={\langle {B}^{-}(t){B}^{+}(s)\rangle }_{E}{e}^{i{\omega }_{0}(t-s)}\mathrm{.}$$

One can see the environmental correlation function as well as the corresponding decoherence process of electron spin is fully determined by the distributions of both the hyperfine interaction strength *A*_*k*_ and the Zeeman splitting of nuclear spin *ω*_*k*_. For instance, with semi-classical approximation, the correlation function often follows an exponential decay^19^; while the Si/Ge core/shell structures offer a promising route to realize a truly uniform coupling^31^ (also termed as the “box” model in GaAs QD). It is shown that (see Method),

6 $${\partial }_{t}\tilde{G}(t)=-{\int }_{0}^{t}ds\tilde{f}(t-s)\tilde{G}(s),$$

where $\tilde{G}(t)\equiv G(t){e}^{-iht}$ with *h* ≡ ∑_*k*_(*A*_*k*_)/(2) given by the Overhauser’s field in the total Hamiltonian (1). Note $\tilde{G}\mathrm{(0)}=G\mathrm{(0)}=1$ and $\tilde{f}(t-s)\equiv f(t-s){e}^{-ih(t-s)}$. It is evident by definition that ${\rm{Re}}(\dot{G}/G)={\rm{Re}}(\dot{\tilde{G}}/\tilde{G})$, such that the damping rate of the central system in Eq. (4) is fully determined by Eq. (6).

## Results

We use the fidelity ${\mathcal F} (t)\equiv \sqrt{\langle \psi \mathrm{(0)|}\rho (t)|\psi \mathrm{(0)}\rangle }$ to measure the decoherence dynamics of the central spin, which is found to be

7 $$ {\mathcal F} (t)=\sqrt{|{c}_{0}(t){c}_{0}{\mathrm{(0)|}}^{2}}=|G(t)|=|\tilde{G}(t)|,$$

assuming the total system starts from $|\psi \mathrm{(0)}\rangle ={C}_{0}\mathrm{|0}\rangle \mathrm{|0}{\rangle }_{E}+{c}_{0}\mathrm{(0)|1}\rangle \mathrm{|0}{\rangle }_{E}+{\sum }_{k}{c}_{k}\mathrm{(0)|0}\rangle {I}_{k}^{+}\mathrm{|0}{\rangle }_{E}$. The dynamics of the exact TCL master equation, parameterized by *ε*(*t*) and *γ*(*t*), is determined by the correlation functions $\tilde{f}(t-s)$ for various environments.

### Decoherence dynamics of the central spin

We first consider an exponential decay correlation function

8 $$\tilde{f}(t-s)=\frac{{\rm{\Gamma }}{\gamma }_{0}}{2}{e}^{-{\gamma }_{0}|t-s|},$$

where Γ measures the coupling strength between the system and environment and *γ*_0_ is usually understood as a measure of the memory capacity or non-Markovianity of the environment. Physically, the correlation function corresponds to the spin-bath modelled by the Ornstein-Uhlenbeck (OU) noise^32,33^, characterized by the auto-correlation (memory) time *τ* = 1/*γ*_0_, where *γ*_0_ is the characteristic cutoff frequency. When *γ*_0_ approaches zero, the correlation function and $|\tilde{G}(t)|$ become constant such that the corresponding environment memorizes the whole evolution history of the system between arbitrary two times *t* and *s*. Contrarily, when *γ*_0_ approaches infinity, the correlation function between *t* and *s* becomes proportional to a delta function, such that the environment completely loses its capacity of memory. The OU noise is widely used since the very beginning of spin resonance experiments^34^ as a classical noise approximation or a long time limit for all the quantum noise induced by spin-spin interactions. It is estimated or attained by the spin-echo alike experiments in many scenario, including spin-lattice interaction, spin-crystal defect interaction, and hyperfine interactions of the central spin to the nuclei, etc. With this exponential decay correlation function, the solution of $\tilde{G}(t)$ in Eq. (6) is (via the Laplace transformation)

9 $$\tilde{G}(t)={e}^{-\frac{{\gamma }_{0}}{2}t}[\cosh (\frac{{\gamma }_{0}\chi }{2}t)+\frac{1}{\chi }\,\sinh (\frac{{\gamma }_{0}\chi }{2}t)],$$

where $\chi =\sqrt{(1-2{\rm{\Gamma }}/{\Upsilon }_{0})}$. It is found then the Stark-shift coefficient *ε*(*t*) = 0 and the decay coefficient *γ*(*t*) in the TCL equation (4) is

10 $$\gamma (t)=\frac{2{\rm{\Gamma }}\,\sinh \,(\frac{{\gamma }_{0}\chi }{2}t)}{\chi \,\cosh \,(\frac{{\gamma }_{0}\chi }{2}t)+\,\sinh \,(\frac{{\gamma }_{0}\chi }{2}t)}\mathrm{.}$$

In case of weak coupling Γ < *γ*_0_/2, ${\mathcal F} (t)=\tilde{G}(t)$ and *χ* is taken as a positive number. When Γ → 0 (vanishing system-environment coupling), *χ* → 1 and ${\mathcal F} (t)\to 1$. In case of strong coupling Γ > *γ*_0_/2, *χ* becomes an imaginary number. The fidelity ${\mathcal F}$ will then decay with an oscillating envelope. An interesting observation is that when Γ = *γ*_0_/2, *γ*(*t*) = 2Γ^2^*t*/(1 + Γ*t*) and

11 $$\tilde{G}(t)={e}^{-{\rm{\Gamma }}t}\mathrm{(1}+{\rm{\Gamma }}t)=1-\frac{{{\rm{\Gamma }}}^{2}{t}^{2}}{2}+{\mathscr{O}}({t}^{3})\approx {e}^{-{{\rm{\Gamma }}}^{2}{t}^{2}}\mathrm{.}$$

It means that the probability for the central electron spin staying at the upper level |1〉 decays in a manner of Gaussian function ${e}^{-{{\rm{\Gamma }}}^{2}{t}^{2}}$ in the short time limit, which can be not be described by the conventional spontaneous emission (exponential decay) for a two-level system embedded in a dissipative bosonic bath or a dissipative optical cavity. Most of the above results can *not* be explained by the Weisskopf-Wigner approximation in quantum optics^35^.

**Figure 1:**
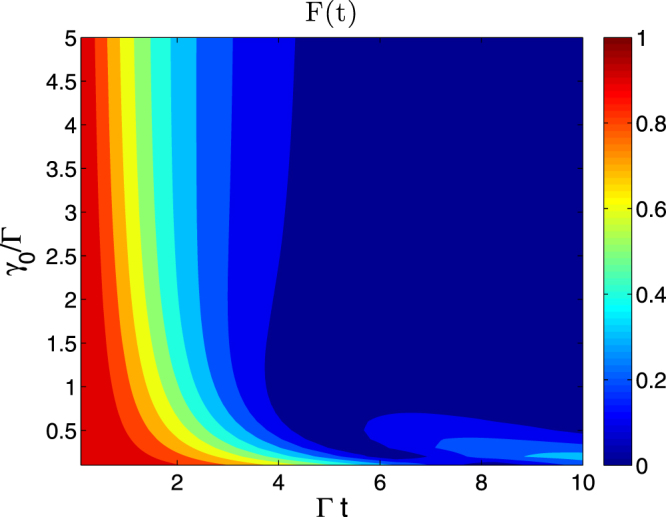
${\mathcal F} (t)=|\tilde{G}(t)|$ in Eq. (9) as a function of dimensionless time Γ*t* and environmental memory parameter *γ*_0_/Γ.

Figure 1 shows the fidelity ${\mathcal F} (t)$ in the space of environmental memory parameter and evolution time. The correlation function is chosen as the exponential decay function implying the OU noise assumption for the spin-bath, which is corresponding to the Lorenz spectral function,

12 $$S(\omega )=\frac{1}{2\pi }\frac{{\rm{\Gamma }}{\gamma }_{0}^{2}}{{\omega }^{2}+{\gamma }_{0}^{2}}\mathrm{.}$$

Then the half-width of the noise spectral is *γ*_0_. Since realistic spin-bath spectra are distributed over a relatively narrow frequency regime, this correlation function is not an accurate characterization but an approximation. Therefore the spectra must be truncated based on physical consideration, and in our evaluation the truncated criterion is set as *γ*_0_/Γ = 5, as shown in the Fig. 1. It is interesting to note that when *γ*_0_/Γ is below 1.0, the fidelity has a revival, which means the quantum information flow (about the initial state of the system) bounces from the spin bath back to the system. This is a typical non-Markovian dynamics. Another interesting phenomenon is that although in the short time limit, the decay speed of fidelity increases with *γ*_0_, whereas the moment when the system spin completely loses its coherence is independent of the memory parameter. By observing the boundary between the light blue and dark blue areas, which implies the lifetime of the fidelity, in Fig. 1, it is evident that when *γ*_0_/Γ < 1, the fidelity lifetime decreases with *γ*_0_. Moreover the fidelity lifetime reaches its maximum value around *γ*_0_/Γ = 1, and then declines asymptotically to a steady value when *γ*_0_/Γ > 1.

We now consider the case where each nuclear spin shares the same hyperfine coupling strength with the central spin and their Zeeman splittings are almost the same, i.e., ${A}_{k}\approx {\mathscr{A}}/N$ and *ω*_*k*_ ≈ *ω*, where *N* is the number of environmental spins. In this model, we have the Overhauser’s field $h={\mathscr{A}}\mathrm{/2}$, and approximately the correlation function is

13 $$\tilde{f}(t-s)\approx \frac{{{\mathscr{A}}}^{2}}{4N}{e}^{i{\rm{\Omega }}(t-s)},\quad {\rm{\Omega }}={\omega }_{0}-\omega -\frac{{\mathscr{A}}}{2}(1-\frac{1}{N})\mathrm{.}$$

Inserting it into Eq. (6), one can find the solution of the propagator is

14 $$\tilde{G}(t)=\frac{{\rm{\Delta }}-{\rm{\Omega }}}{2{\rm{\Delta }}}{e}^{i({\rm{\Omega }}+{\rm{\Delta }})t\mathrm{/2}}+\frac{{\rm{\Delta }}+{\rm{\Omega }}}{2{\rm{\Delta }}}{e}^{i({\rm{\Omega }}-{\rm{\Delta }})t\mathrm{/2}},$$

where ${\rm{\Delta }}\equiv \sqrt{{{\rm{\Omega }}}^{2}+{{\mathscr{A}}}^{2}/N}$. The system will periodically come back to its initial state when the absolute value of $|{c}_{0}(t)|=|\tilde{G}(t)|=1$. Note in this case,

15 $$|\tilde{G}(t)|=|\cos \,\frac{{\rm{\Delta }}t}{2}-i\frac{{\rm{\Omega }}}{{\rm{\Delta }}}\,\sin \,\frac{{\rm{\Delta }}t}{2}|\mathrm{.}$$

Now we can see a periodical recoherence phenomenon at the moments Δ*t* = 2*nπ* where *n*’s are arbitrary integers. This “box” model, as an extreme non-Markovian case, describes that the central spin coherence can be transferred back and forth with a collective state of the surrounding environmental spins, and then allows for exploiting the environmental spins to store quantum state of the central spin. In this situation, the leading decoherence mechanism for the stored state is bath-spin diffusion, with dephasing rates in the kHz domain. Techniques similar to spin-echo^36,37^ can be used to mitigate the pure dephasing effect.

Solid (semiconductor) spin-based qubits are promising candidates for quantum computation because of their scalability. The fundamental single-qubit gates have been demonstrated for GaAs-based spin qubits^38^. The typical data of the III-V semiconductor compounds for quantum dot^38^ show that $|{\omega }_{0}|\approx |{\mathscr{A}}|\approx {\mathrm{(10}}^{2}\sim {10}^{3})|{\omega }_{k}|\approx {\mathrm{(10}}^{4}\sim {10}^{6})|{A}_{k}|$. It means that the difference between upper and lower bounds of *A*_*k*_ can be ignored for the magnitude of *A*_*k*_ is at least four-order smaller than that of the system frequency *ω*_0_. As for the nuclear spin frequencies *ω*_*k*_, their magnitudes are three-order smaller than *ω*_0_. The magnitude separation for these three physical parameters implies that the “box” model is a reasonable idealization. The inherent error caused by the hyperfine coupling therefore features with a naturally strong non-Markovian character and then can be controlled to some extent. The Overhauser’s effect is determined by the signs of *A*_*k*_ or *h* relative to *ω*_0_. If they share the same sign, Ω will then be reduced. This is equivalent to reduce the energy splitting of the central spin and makes it more fragile to the decoherence induced by flip-flop term. Otherwise, the Overhauser’s field could naturally protect the coherence of the central spin.

Let us now consider a more realistic situation where the hyperfine interaction strength *A* are *k*-dependent, then the correlation function can be expressed as

16 $$\tilde{f}(t,s)=\sum _{k}\frac{{A}_{k}^{2}}{4}{e}^{i{{\rm{\Omega }}}_{k}(t-s)},$$

where Ω_*k*_ = *ω*_0_ − *ω*_*k*_ − *A*_*k*_/2. As discussed in the “box” model, it is reasonable to assume that ${A}_{k}\approx {\mathscr{A}}/N$ and *ω*_*k*_ satisfies a Gaussian distribution characterized by the mean value $\bar{\omega }$ and the variance *ν*^2 ^^30^, where $\bar{\omega }$ and *ν* can be supposed to be in the same order of $|{\mathscr{A}}|/\sqrt{N}$^30,38^. In reality ${\omega }_{0}\approx {\mathscr{A}}=N{A}_{k}\approx \sqrt{N}{\omega }_{k}$ and are much larger than *ω*_*k*_, which can be approximated as a continuous variable centering around the average value $\sim {\mathscr{A}}/\sqrt{N}$ following Gaussian distribution:

17 $$P(\omega )=\frac{1}{\sqrt{2\pi }\nu }{e}^{-\frac{{(\omega -\bar{\omega })}^{2}}{2{\nu }^{2}}}\mathrm{.}$$

So that it is reasonable to have Ω_*k*_ ≈ *A* − *ω* in Eq. (16), then the effective correlation function of the spin bath can then be evaluated as

18 $$\tilde{f}(t-s)\approx \frac{{{\mathscr{A}}}^{2}}{4N}{e}^{i{\mathscr{A}}(t-s)}\int d\omega P(\omega ){e}^{-i\omega (t-s)}\approx \frac{{{\mathscr{A}}}^{2}}{4N}{e}^{-\frac{{\nu }^{2}}{2}{(t-s)}^{2}+i{\mathscr{A}}(t-s)},$$

which is insensitive to the mean frequency $\bar{\omega }$ of the bath-spins (${A}_{k}\ll {\omega }_{k}\ll {\omega }_{0}\sim {\mathscr{A}}$). The fidelity ${\mathcal F} (t)=|\tilde{G}(t)|$ can be numerically obtained by inserting Eq. (18) into Eq. (6).

**Figure 2:**
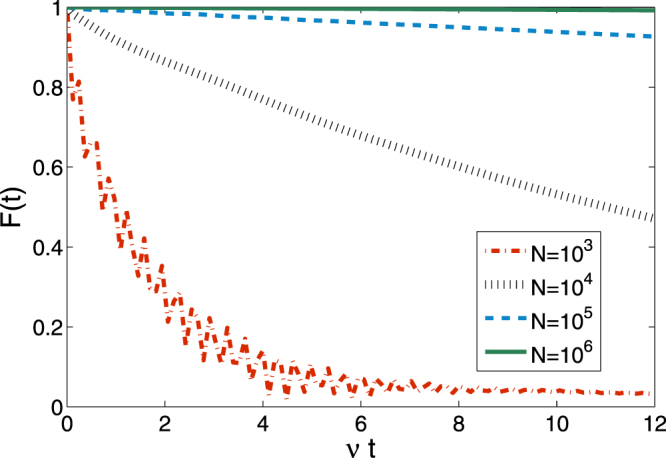
${\mathcal F} (t)=|\tilde{G}(t)|$ as a function of the dimensionless time *νt*, where *ν* is the variance of *ω*_*k*_ supposed to be equivalent to the average of *ω*_*k*_, and *N* is the number of bath-spins.

Figure 2 demonstrates ${\mathcal F} (t)$ as a function of the number of environmental spins *N*. This simulation is performed for the quantum dot system based on III-V semiconductor compounds. By using the experimental data listed in ref.^30^, for different number of nuclear spins we fix the summation and the variance of coupling strength between the electron spin and nuclear spin as ${\mathscr{A}}={10}^{2}\bar{\omega }$ and $\nu =\bar{\omega }$, respectively, where the mean frequency of bath-spin $\bar{\omega }$ is used as an energy unit. Under these constrains, one can see that qualitatively the coupling strength *A*_*k*_ of each nuclear spin to the central spin decreases with the number of nuclear spin *N*. It is interesting to compare the decoherence patterns for different *N*. When *N* = 10^3^, the fidelity decays with a moderate fluctuation, meaning that the exchange of the quantum information and energy between the system and bath spins is significant. When *N* increases, the amplitude of fluctuation vanishes and the decay rate of the central spin becomes gradually smaller. As such the coherence of the central spin remains robust for large *N*. The spin-bath with *N* up to 10^6^ works as similar as that in the “box” model on providing a natural protection for central spin coherence. Qualitatively, our result (pattern) shown in Fig. 2 agrees with that obtained by the perturbative estimation on the decoherence of a solid-state electron spin qubit in III-V semiconductor QDs coupled by long-range hyperfine-mediated interactions^30^ with different number of nuclear spins.

### Eliminating the leakage induced by the spin-environment

We have seen the unique features that some spin baths have self-protection of the central spin coherence. Now we come to discuss how to actively defeat the decoherence induced by spin baths with Lorentz spectra or modelled by the OU noise (8). The open-loop quantum control strategy, leakage-elimination operator, uses fast and strong “bang-bang” (BB) pulses^27^ and decomposes a general total Hamiltonian as *H*_tot_ = *H*_*P*_ + *H*_*Q*_ + *L*. Here *H*_*P*_ (*H*_*Q*_) lives in the interested subspace (the subspace orthogonal to *H*_*P*_) and *L* represents the couplings between the subspaces *P* and *Q*, which in general induce the leakage of the quantum states in *P*-subspace into those in *Q*-subspace. Any operator *R*_*L*_ satisfying

19 $$\{{R}_{L},L\}=\mathrm{0,}\quad [{R}_{L},{H}_{P}]=\mathrm{0,}\quad [{R}_{L},{H}_{Q}]=\mathrm{0,}$$

can serve as a leakage-elimination operator. It is verified that *R*_*L*_ does not affect the evolution determined by *H*_*P*_ and *H*_*Q*_, however, ${R}_{L}^{\dagger }L{R}_{L}=\,-L$. Thus using a BB parity-kick sequence, it follows that *R*_*L*_ eliminates the effect by the unwanted Hamiltonian *L*, which represents but is not restricted to the system-bath interaction,

20 $$\mathop{\mathrm{lim}}\limits_{m\to \infty }{({e}^{-i{H}_{{\rm{tot}}}t/m}{R}_{L}^{\dagger }{e}^{-i{H}_{{\rm{tot}}}t/m}{R}_{L})}^{m}={e}^{-i{H}_{Q}t}{e}^{-i{H}_{P}t}\mathrm{.}$$

When prepared in *P*-subspace, the system will be well-protected. The original LEO method is similar to the widely-used dynamical decoupling (DD) methods^39,40^. All of them are based on the Magnus expansion and Trotter-Suzuki expansion. Typically, periodic DD could be used to correct the first-order error in the Magnus expansion. More specifically, if the control Hamiltonian is described by *H*_*c*_ = *Jσ*_*z*_, then the evolution operator combining the control mechanism becomes *U*(*δ*_*t*_) = exp(−*iH*_tot_*δ*_*t*_ − *iJσ*_*z*_*δ*_*t*_) ≈ −*iσ*_*z*_ when *Jδ*_*t*_ = *π*/2 in a short period of time *δ*_*t*_. Under this condition, the control strength *J* goes to infinity when *δ*_*t*_ goes to zero. Although more advanced DD, e.g., concatenated DD, is used to correct the error cumulated by PDD, yet without comparison with exact (numerical or analytical) solutions, it is difficult to know the valid range of the zeroth-order perturbation or higher-order perturbation theory for a physical model with realistic system-bath interactions. Additionally, these perturbative expansions always have risks of divergence. Note most of the control approaches are based on the master equations or equivalent equations with the standard Born-Markovian approximation. Therefore, it is reasonable to extend the standard perturbative dynamical coupling theory to a more general domain where nonideal pulses can be employed in the nonperturbative control process.

It has been found that the parity-kick sequence can be relaxed to ${R}_{L}\Rightarrow {U}_{L}(t)={e}^{-i{\int }_{0}^{t}ds{H}_{L}(s)}$, with *H*_*L*_(*t*) = *r*(*t*)*R*_*L*_. Alternatively, it is equivalent to add a control operator *H*_*L*_(*t*) into the system Hamiltonian, where *r*(*t*) is a time-dependent control function. Then the original LEO can be extended into a nonperturbative version, as we have done recently^29^. A distinguishing character for *H*_*L*_(*t*) is, as shown in the pervious and present studies (See ref.^29^ and the references therein), that *r*(*t*) can be almost any sequences such as period sequences with arbitrary shapes and chaotic and even noisy signals, as long as the control is sufficiently strong and fast-oscillating.

The control approach can be immediately applied to strengthen robustness of the central spin, in particular eliminating the effect from the flip-flop terms in Eq. (1) or Eq. (2). Specifically, LEO for this system is found to be *R*_*L*_ = *S*_*z*_, which means that in QD experiments one can fluctuate the energy splitting of the electron spin by external magnetic field. Consequently the corresponding control Hamiltonian is *H*_*L*_ = *r*(*t*)*S*_*z*_ that is used to interpolate the free evolution of the whole system. By using the fast bang-bang pulse sequence, one can verify that

21 $$\mathop{\mathrm{lim}}\limits_{m\to \infty }{({e}^{-i\frac{{H}_{{\rm{tot}}}^{I}t}{m}}{R}_{L}^{\dagger }{e}^{-i\frac{{H}_{{\rm{tot}}}^{I}t}{m}}{R}_{L})}^{m}={e}^{-i\mathrm{|1}\rangle \langle \mathrm{1|}{B}^{z}t},$$

i.e., the decoherence effect by the flip-flop terms of the hyperfine interaction has been dynamically corrected. Here we make use of the anticommutator and the commutator

22 $$\{{R}_{L},{S}_{-}(t){B}^{\dagger }(t)+h\mathrm{.}c\mathrm{.\}}=\mathrm{0,}\quad [{R}_{L}\mathrm{,|1}\rangle \langle \mathrm{1|}{B}^{z}]=\mathrm{0,}$$

and the Trotter formula that the evolution operator determined by Eq. (2)

23 $$U(t)={{\mathscr{T}}}_{\to }({e}^{-i{\int }_{0}^{t}ds{H}_{{\rm{tot}}}^{I}})=\mathop{\mathrm{lim}}\limits_{m\to \infty }{({e}^{-i\frac{{H}_{{\rm{tot}}}^{I}t}{m}})}^{m}\mathrm{.}$$

This result holds to the order of *t*^2^ only and is an idealization or perturbation. In the case of nonperturbative LEO, the coupling *L* will modify the correlation function $\tilde{f}(t-s)$ into

24 $$g(t-s)=\tilde{f}(t-s){e}^{-i{\int }_{s}^{t}ds^{\prime} r(s^{\prime} )}=\tilde{f}(t-s){e}^{-iR(t-s)},$$

where $R\equiv \frac{{\int }_{s}^{t}ds^{\prime} r(s^{\prime} )}{t-s}$. According to the Riemann-Lebesgue lemma, since the maximal frequency of the function $|\tilde{f}(t-s)\tilde{G}(s)|$ has a finite upper bound, thus the integral in Eq. (6)

25 $${\int }_{0}^{t}dsg(t-s)\tilde{G}(s)\propto {\int }_{0}^{t}{e}^{iRs}\tilde{f}(t-s)\tilde{G}(s),$$

approaches zero as *R* → ∞. Therefore when $\tilde{R}(t)\equiv {\int }_{0}^{t}dsr(s)$ is sufficient large or the oscillation of ${e}^{i\tilde{R}(t)}$ is sufficient fast, one can use LEO to suppress the decoherence of the system spin. Consequently and generally, the effectiveness of LEOs depends solely on the integral ${\int }_{s}^{t}ds^{\prime} r(s^{\prime} )$ in the time domain but not the details of *r*(*t*).

**Figure 3:**
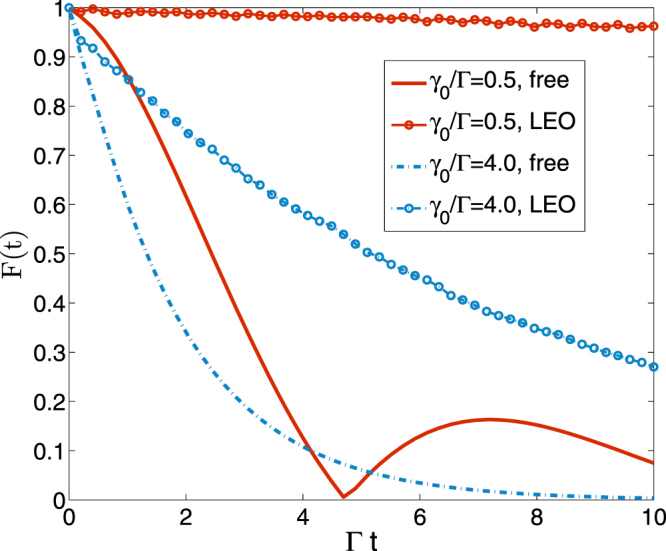
The free and controlled dynamics of ${\mathcal F} (t)=|\tilde{G}(t)|$ as a function of dimensionless time Γ*t* with different environmental memory parameter *γ*_0_. The spin-bath we employed here follows the correlation function described in Eq. (8). In the control dynamics by LEO protocol, we apply a random pulse sequence, whose period and duration time are *τ* = 0.02Γ*t* and *κ* = 0.01Γ*t*, respectively, and the average strength is Ψ = 0.2Γ.

Figure 3 demonstrates the free and controlled dynamics of the fidelity ${\mathcal F} (t)$. For the LEO control protocol applied in this work, we use an equiv-distant rectangular pulse sequence but with randomized control strength in each period, whose intensity is set as rand(*t*)Ψ/*κ* in the duration time *κ* and as zero in the dark time for each period *τ*, where rand(*t*) is a random number between zero and one and Ψ is the average strength. It is important to emphasize that the LEO control works excellently in a strong non-Markovian regime characterized by 1/*γ*_0_ but less functional in the nearly Markovian regime. Therefore LEO is a reliable tool to decouple the central spin from influence of the spin bath in non-Markovian regime. It should be emphasized that our formalism also clarifies an unconventional fact that *an ideal Markovian process cannot be controlled via external fields* because in the Markovian limit $\tilde{f}(t-s)\propto \delta (t-s)$, any control factor *e*^*iRs*^ in Eq. (24) becomes useless. One can see from Eq. (6) that the propagator as well as the fidelity of the central system state would experience an irreversibly monotonic decay. In other words, a Markovian dynamics cannot be affected by external controlled fields.

## Discussion

In this paper, we have proposed an exact master equation for a central spin coupled to a spin bath by hyperfine interaction, which represents a family of physical spin-bath models ubiquitously existing in semiconductor systems. We analyze the decoherence dynamics determined by the spin baths modelled by Ornstein-Uhlenbeck noise, uniform hyperfine interaction strength and Gaussian distribution in terms of bath-spin frequency, respectively. Described by the OU noise, the effect of the spin bath on the central spin gives rise to a colorful reduced dynamics that is beyond the scope of Weisskopf-Wigner approximation. We have found that the Overhauser’s field in QD system may help to suppress the decoherence process of the central electron spin, which can regain its coherence in a periodic pattern under the uniform coupling approximation and retain its initial state under an environment with a larger number of bath spins but with an invariant sum of coupling strengths.

More importantly, the existence of the exact master equation (4) allows us to apply the nonperturbative leakage-elimination operator to protect the quantum coherence of the central spin. Since any approximate master equation may introduce additional uncertainty such that one cannot justify if the control effectiveness is from control itself or from the artificialness of the approximation, it is absolutely necessary to have an exact master equation, as a standard tool, to study the dynamics and control in the same manner. Our exact master equation provides such a trustworthy tool.

## Method

We suppose that the total system starts from

26 $$|\psi \mathrm{(0)}\rangle ={C}_{0}\mathrm{|0}\rangle \mathrm{|0}{\rangle }_{E}+{c}_{0}\mathrm{(0)|1}\rangle \mathrm{|0}{\rangle }_{E}+\sum _{k}{c}_{k}\mathrm{(0)|0}\rangle {I}_{k}^{+}\mathrm{|0}{\rangle }_{E},$$

where the subscript *E* indicates the base of spin environment. The time evolved state can be written by the following expression with undetermined amplitudes,

27 $$|\psi (t)\rangle ={C}_{0}\mathrm{|0}\rangle \mathrm{|0}{\rangle }_{E}+{c}_{0}(t\mathrm{)|1}\rangle \mathrm{|0}{\rangle }_{E}+\sum _{k}{c}_{k}(t\mathrm{)|0}\rangle {I}_{k}^{+}\mathrm{|0}{\rangle }_{E},$$

which is obtained by the Hamiltonian in Eq. (2) and the Schrödinger equation ${\partial }_{t}|\psi (t)\rangle =\,-i{H}_{{\rm{tot}}}^{I}|\psi (t)\rangle$ represented in the single-exciton subspace. It is straightforward to see the amplitude *C*_0_ of the vacuum base |0〉|0〉_*E*_, will remain constant in the evolution. While the other amplitudes satisfy

28 $$\frac{d}{dt}{c}_{0}(t)=ih{c}_{0}(t)-i\sum _{k}\frac{{A}_{k}}{2}{e}^{i({\omega }_{0}-{\omega }_{k}+\frac{{A}_{k}}{2})t}{c}_{k}(t),$$

29 $$\frac{d}{dt}{c}_{k}(t)=-i\frac{{A}_{k}}{2}{e}^{-i({\omega }_{0}-{\omega }_{k}+\frac{{A}_{k}}{2})t}{c}_{0}(t),$$

where *h* ≡ ∑_*k*_(*A*_*k*_)/(2). Here we assume the electron spin is initially at the upper state |1〉 under the magnetic field and the spin bath is in a fully polarized state, *c*_*k*_(0) = 0, as done in ref.^16^. This initial condition is evidenced by recent high polarization experiments in quantum hall edge states^41^ ($\sim \mathrm{85 \% }$) and a bias voltage in a ballistic quantum wire^42^ ($\sim \mathrm{94 \% }$). Additionally, the fully polarized nuclei have been proposed as the storage of an electron spin state^19^. Therefore the coefficient *c*_*k*_(*t*) can be formally written as

30 $${c}_{k}(t)=-i\frac{{A}_{k}}{2}{\int }_{0}^{t}ds{e}^{-i({\omega }_{0}-{\omega }_{k}+\frac{{A}_{k}}{2})s}{c}_{0}(s\mathrm{).}$$

Substituting it into Eq. (28), we obtain an exact time-convolution dynamical equation for the central spin,

31 $$\frac{d}{dt}{c}_{0}(t)=ih{c}_{0}(t)-{\int }_{0}^{t}dsf(t-s){c}_{0}(s),$$

where the kernel function is given by a two-point correlation function of the reservoir

32 $$f(t-s)=\sum _{k}{(\frac{{A}_{k}}{2})}^{2}{e}^{i({\omega }_{0}-{\omega }_{k}+\frac{{A}_{k}}{2})(t-s)}\mathrm{.}$$

We now define a propagator *G*(*t*) that

33 $${c}_{0}(t)=G(t){c}_{0}\mathrm{(0),}$$

which does not depend on the initial condition due to the convex-linear characteristic in the decomposition of system initial state. Furthermore, we define $\tilde{G}(t)\equiv G(t){e}^{-iht}$ and can show, by inserting it into Eq. (31), that it satisfies ${\partial }_{t}\tilde{G}(t)=-{\int }_{0}^{t}ds\tilde{f}(t-s)\tilde{G}(s)$. To construct an exact master equation for the central spin in a time-convolutionless form, i.e., ${\partial }_{t}\rho (t)={{\mathscr{K}}}_{{\rm{TCL}}}(t)\rho (t)$, one can use an exact dynamical map Φ(*t*), which transforms the initial states into the states at time *t*: *ρ*(*t*) = Φ(*t*)*ρ*(0), i.e., ${{\mathscr{K}}}_{{\rm{TCL}}}(t)=\dot{{\rm{\Phi }}}(t){{\rm{\Phi }}}^{-1}(t)$. The density matrix for the central spin *ρ*(*t*) is obtained by Tr_E_[|*ψ*(*t*)〉〈*ψ*(*t*)|]. Then due to Eq. (27), it is expressed as

34 $$\rho (t)=(\begin{array}{cc}{\rho }_{11} & {\rho }_{10}\\ {\rho }_{01} & {\rho }_{00}\end{array})=(\begin{array}{cc}|{c}_{0}(t{)|}^{2} & {C}_{0}^{\ast }{c}_{0}(t)\\ {C}_{0}{c}_{0}^{\ast }(t) & 1-|{c}_{0}(t{)|}^{2}\end{array})\mathrm{.}$$

Consider the time derivative of *ρ*(*t*)

35 $${\partial }_{t}\rho (t)=(\begin{array}{cc}{\rm{R}}e(\frac{{\dot{c}}_{0}}{{c}_{0}})|{c}_{0}(t{)|}^{2} & (\frac{{\dot{c}}_{0}}{{c}_{0}}){C}_{0}^{\ast }{c}_{0}(t)\\ (\frac{{\dot{c}}_{0}^{\ast }}{{c}_{0}^{\ast }}){C}_{0}{c}_{0}^{\ast }(t) & -{\rm{R}}e(\frac{{\dot{c}}_{0}}{{c}_{0}})|{c}_{0}(t{)|}^{2}\end{array}),$$

the commuter

36 $$[{S}_{z},\rho (t)]=(\begin{array}{cc}0 & {\rho }_{10}\\ -{\rho }_{01} & \mathrm{0,}\end{array}),$$

the Lindbladian

37 $$[{S}_{-}\rho (t){S}_{+}-\frac{1}{2}\{{S}_{+}{S}_{-},\rho (t)\}]=(\begin{array}{cc}-{\rho }_{11} & -\frac{{\rho }_{10}}{2}\\ -\frac{{\rho }_{01}}{2} & {\rho }_{11}\end{array}),$$

and the relation ${\dot{c}}_{0}/{c}_{0}=\dot{G}/G$ due to Eq. (33), we can obtain an exact TCL master equation in the interaction picture

38 $${\partial }_{t}\rho (t)=-\frac{i}{2}\varepsilon (t)[{S}_{+}{S}_{-},\rho (t)]+\gamma (t)[{S}_{-}\rho (t){S}_{+}-\frac{1}{2}\{{S}_{+}{S}_{-},\rho (t)\}],$$

where $$\varepsilon (t)\equiv -2{\rm{Im}}[\dot{G}(t)/G(t)]$$ and $$\gamma (t)\equiv -2{\rm{Re}}[\dot{G}(t)/G(t)]$$.
